# Analysis of Five Earthy-Musty Odorants in Environmental Water by HS-SPME/GC-MS

**DOI:** 10.1155/2014/697260

**Published:** 2014-01-02

**Authors:** Zhen Ding, Shifu Peng, Weiwen Xia, Hao Zheng, Xiaodong Chen, Lihong Yin

**Affiliations:** ^1^School of Public Health, Southeast University, Nanjing, Jiangsu 210009, China; ^2^Department of Environmental and Endemic Diseases Control, Jiangsu Center for Disease Control and Prevention, Nanjing, Jiangsu 210009, China; ^3^Department of Physical and Chemical Test, Jintan Center for Disease Control and Prevention, Changzhou, Jiangsu 213200, China

## Abstract

The pressing issue of earthy and musty odor compounds in natural waters, which can affect the organoleptic properties of drinking water, makes it a public health concern. A simple and sensitive method for simultaneous analysis of five odorants in environmental water was developed by headspace solid-phase microextraction (HS-SPME) coupled to chromatography-mass spectrometry (GC-MS), including *geosmin* (GSM) and 2-methylisoborneol (2-MIB), as well as dimethyl trisulfide (DMTS), **β**-cyclocitral, and **β**-ionone. Based on the simple modification of original magnetic stirrer purchased from CORNING (USA), the five target compounds can be separated within 23 min, and the calibration curves show good linearity with a correlation coefficient above 0.999 (levels = 5). The limits of detection (LOD) are all below 1.3 ng L^−1^, and the relative standard deviation (%RSD) is between 4.4% and 9.9% (*n = 7*) and recoveries of the analytes from water samples are between 86.2% and 112.3%. In addition, the storage time experiment indicated that the concentrations did not change significantly for GSM and 2-MIB if they were stored in canonical environment. In conclusion, the method in this study could be applied for monitoring these five odorants in natural waters.

## 1. Introduction

Earthy and musty odors in drinking water are often associated with the metabolites which are produced in the degradation of cyanobacteria, actinomyces, fungi, and blue-green algae [1–3], including geosmin (GSM) and 2-methylisoborneol (2-MIB), commonly found in lakes and reservoirs [4, 5]. Moreover, attention now is drawn to the compounds dimethyl trisulfide (DMTS), **β**-cyclocitral, and **β**-ionone, which are also associated with algal blooms caused by eutrophication progress [6–9], and they often simultaneously break out in environmental waters [4, 10]. *Beta-ionone*, for instance, potentially derived from carotenoids, is the significant component of flavor and aroma in some fruits and vegetables [11, 12]. In studies conducted according to the SIDS initial assessment report [13], **β*-ionone* has only low acute toxicity after oral ingestion by animal experiments and none of volunteers showed a positive reaction. More specifically, the two main exposures, occupational exposure may occur during manufacture and industrial using, which is the skin contact and inhalation and is limited by enclosed systems and personal protective measures, as well as consumer exposure in food and some house wares which is also low since small amounts around 5 ppm (parts per million) in food and at usual concentrations of up to 0.3% in cosmetics. However, the odor threshold concentration (OTC) is extremely low, 10 ng L^−1^ or less for GSM and 2-MIB [14], for instance, which can be detected by human nose. The low threshold of detection can result in consumer complaints about the terrible malodors in recreational waters, aquatic products, and tap water, especially during the outbreak period of algal blooms [8, 15, 16], even if some other quality indicators of water, such as turbidity, number of algal cells, and suspended matter, are acceptable. Therefore, the identification and quantification of these trace compounds are essential since they dramatically influence the esthetic quality and consumer acceptability of drinking water.

For now, a variety of techniques have been established and applied for enrichment and extraction of earthy and musty compounds. Among these techniques, closed-loop stripping analysis (CLSA) and some of its modified versions have been widely used for trace odorants such as GSM and 2-MIB in water samples. The result showed that CLSA was a good tool for analysis of GSM and 2-MIB at a low level [17]. Some other methods such as purge and trap (P&T) coupled to gas chromatography with mass spectrometry [18, 19] or to GC-FID [20], liquid-liquid microextraction (LLME) [21], stir bar sorptive extraction (SBSE) [22–24], and solid-phase extraction (SPE) [25] can also be taken to detect the earthy and musty odors in water at nanogram-per-liter level. Although these techniques greatly improve the limits and sensitivity of detection, some shortcomings restrict extensive usage of these methods, including unsuitable for the analysis of low-boiling-point odors and time-consuming (SPE, SBSE) [26, 27], lacking stability of droplet during extraction (LLME), and the sodium chloride, could be spurge onto the upside of purge tube and subsequently the sodium chloride was dragged in tubes and valves, causing abrasion by using P&T [10, 18, 28]. As technology advances, solid phase microextraction (SPME) was first developed and reported that headspace SPME (HS-SPME) was effective for collecting volatile organic compounds from Penicillium [29]. HS-SPME has become one of the most popular techniques in pretreating and enriching the odorants in water [30–34], because of no solvent during extraction by HS-SPME which cannot be achieved by LLME and simpler operation when comparing other methods like as SPE, CLSA, and SBSE, and the most important merit is that the targets can be enriched selectively by suitable fiber, which cannot be obtained by SPE and LLME. There are few reports regarding the HS-SPME to detect five or more odor compounds simultaneously in water samples, and some reports limited to two common odors as GSM and 2-MIB [31, 33–35]. However, the noteworthy is that their study indicated that the HS-SPME had excellent performance in studying trace odors in natural waters.

This study details a simple and sensitive method for simultaneous analysis of five odors in environmental water by using HS-SPME coupled to GC-MS, including GSM and 2-MIB, as well as DMTS, **β**-cyclocitral, and **β**-ionone. The proposed method has been validated by variables on the five compounds, such as limit of detection (LOD), recovery, measurement precision (%RSD), and it also has been applied to environmental waters. In addition, the storage time experiment indicated that the concentrations did not change significantly for both GSM and 2-MIB if they were stored in canonical environment in ten days.

## 2. Materials and Methods

### 2.1. Chemicals, HS-SPME Apparatus, and Samples

The six standard compounds, GSM, 2-MIB, **β**-cyclocitral, and *2*-isobutyl-3-methoxypyrazine (IBMP, as the internal standard) were obtained from Sigma-Aldrich (100 mg L^−1^ in methanol); DMTS and **β**-ionone were also purchased from Sigma-Aldrich in the highest purity available. One mg L^−1^ mixed stock standard solutions of five target compounds was prepared in methanol, and all of them were stored in the dark at 4°C. The details of the six compounds are shown in Table 1.

Deionized water was prepared on a water purification system (Gradient A10) supplied by Millipore (Billerica, MA, USA). Sodium chloride (analytical grade, China), which was added to the samples before extraction, was conditioned by heating at 450°C for 4 h before use. SPME apparatus was purchased from Supelco (USA), including fiber DVB/CAR/PDMS, PMDS/DVB and PMDS, fiber holder, sampling stand, magnetic stirrer, injection catheter, and 60 mL specialized vials for SPME.

Water samples from three waterworks in Wuxi city *(120:18E-31:35N)* were analyzed by using the proposed method, one source water, one product water, and one tap water were collected from each waterworks, nine samples in total. Water samples were filtered through 0.45 *μ*m glass-fiber-filter (GF/C, Whatman, England) if necessary and kept in 350 mL sample vials with PTFE-faced silicone septum and stored at 4°C before analysis.

### 2.2. SPME Procedures

After putting NaCl and a stir bar in a 60 mL vial, 40 mL of mixed standard solutions or environmental water samples was added, and IBMP (20 ng L^−1^ in 40 mL water sample) was added to every sample when using internal standard method. The vial was sealed with PTFE septum cap and placed in a water bath. Several minutes after the temperature was achieved in the vial, the outer needle of fiber was used to penetrate the septum and the fiber was exposed to the headspace for extraction. After exposure, the fiber was immediately inserted into GC injection port for desorption.

### 2.3. Gas Chromatography-Mass Spectrometry

A Varian 300 GC/MS/MS (Varian Inc. CA, USA) with ion trap and mass spectrometer was obtained with a Varian VF-5 MS capillarity column (30 m × 0.25 mm × 0.5 *μ*m). The temperature of the injector was 230°C and adjusted to splitless mode at the eighth minute. The carrier gas was helium at a flow of 1 mL min^−1^. The temperature of the oven started at 40°C and was held for 5 min. Then the temperature was 8°C min^−1^ to achieve 160°C (total time 20 min) followed by 20°C min^−1^ to achieve 260°C (25 min in total). The electron impact (EI)-MS conditions were as follows: ion-source temperature, 230°C; MS transfer line temperature, 250°C; solvent delay time, 5 min; ionizing voltage, 70 eV. The mass spectrogram in full scan mode was obtained at the *m*/*z* range of 60–260. According to the MS scan function (SIM mode), the process was divided into six main segments as shown in Table 2. The method of internal standard [31, 33] was applied to construct calibration curve and determine concentrations of five odorants in water.

## 3. Results and Discussion

### 3.1. Improvements in HS-SPME Apparatus

**Figure 1:**
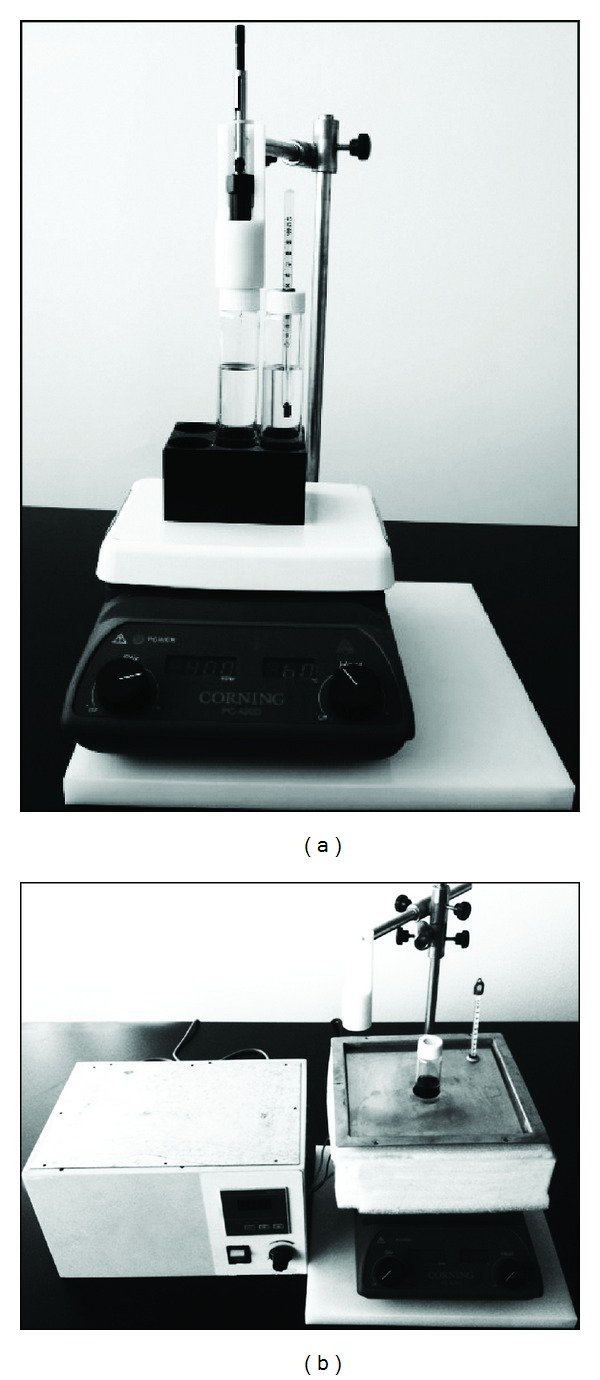
(a) The original magnetic stirrer from CORNING and (b) modified one.

The HS-SPME apparatus was obtained from Supelco, as shown in Figure 1(a). The original apparatus has some demerits in practice, which can be classified as follows: firstly, it would take relatively long time to reach or adjust the proposed temperature, especially in low environmental temperature such as in winter, because the body of sample vial is almost fully exposed to the environment and difficult to keep a stable temperature; secondly, the temperature of sample or the extraction is recorded by the thermometer in adjacent vial, and this is not reliable or it cannot guarantee the same temperature in both of them since the two vials are independent of each other in respective dynamic system due to uneven heating and natural air flow. However, some studies [31, 33, 35] had never addressed the above issues. Therefore, we tried to transform the original apparatus into a novel one. As shown in Figure 1(b), the digital magnetic stirrer was retained to obtain accurate and comparable values which can contrast with other peer reports. However, we apply the thermostat water bath to control the vial temperature freely, and it can be quickly and accurately adjusted to proposed temperature if we study the effect of the extraction temperature, which can efficiently overcome the weak points above and put its merits into full use.

### 3.2. Selection of the Fiber

**Figure 2:**
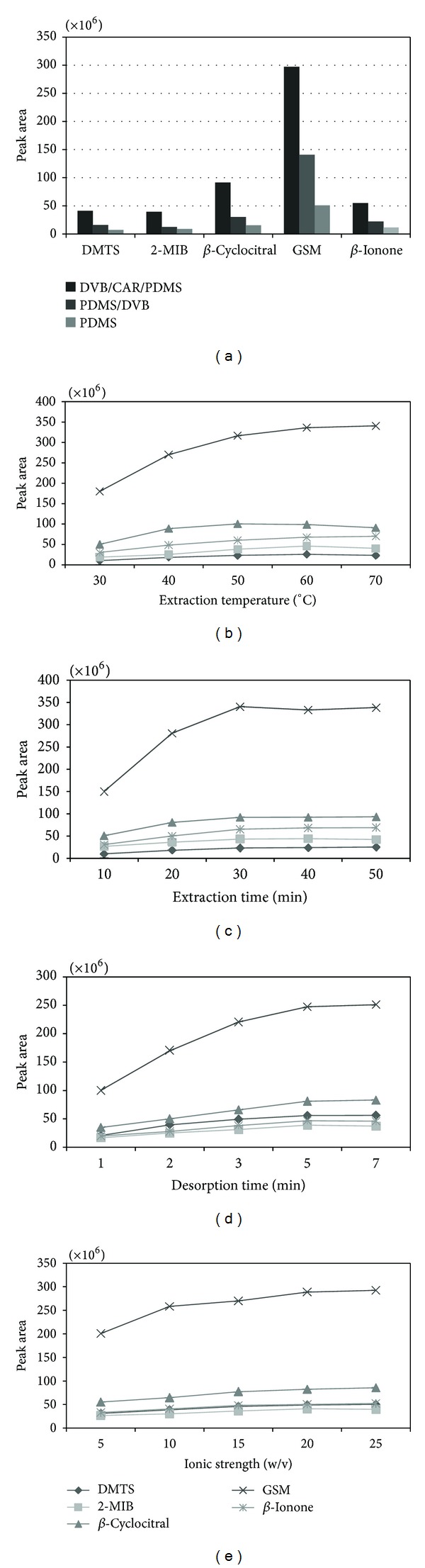
The effect of (a) fiber, (b) extraction temperature, (c) extraction time, (d) desorption time, and (e) ionic strength on the HS-SPME/GC-MS of five target compounds, and 100 ng L^−1^ of mixed standard solutions was analyzed by (a) fiber exposition at 60°C, 30 min, for 25% (w/v) ionic strength, (b) fiber exposition at 25% (w/v) ionic strength for 30 min, (c) fiber exposition at 60°C for 25% (w/v) ionic strength, and (d) and (e) at 60°C for 30 min.

Fiber coatings dominate the effect of extraction or recoveries of analytes. According to the principles of fiber selection from Supelco, that is, the polarity and thickness of the stationary phase coating on the fiber, and also based on the earlier reports [31, 33, 36], three commercial fibers (DVB/CAR/PDMS, PDMS/DVB, and PDMS) were chosen for evaluation in this study.  Figure 2(a) showed the extraction yield of three fibers (expressed by peak area), and it was concluded that DVB/CAR/PDMS fiber extracted almost all of analytes with the best performance. Thus, this coated fiber was chosen in our study and for further experiments.

### 3.3. Effect of Extraction Temperature

As shown in Figure 2(b), we studied the HS-SPME analyses run at a selected temperature. The extraction efficiency of five targeted analytes increased as extraction temperature from 30°C to 60°C, especially sharply increasing between 30°C and 40°C, and slowly growing until 60°C. However, a decrease was observed between 60°C and 70°C for 2-MIB and *β*-cyclocitral. The potential reasons can be as follows: firstly, the increased amount of water vapor would be assembled on the fiber as temperature growing, which would reduce the extraction efficiency; secondly, the different molecular weight of odorants was deemed to be inconsistently susceptive to fiber [37]; thirdly, this can be understood by the partition coefficient between the fiber and analytes. In other words, according to the formula *K*_fs_ = *K*_0_exp[−Δ*H*/*R*(1/*T* − 1/*T*_0_)] [38], the partition coefficient (*K*_fs_) would change if extraction temperature alters from *T*_0_ to *T*, because potential energy of analyte on coating material would be less than that in the sample if the *K*_fs_ value is more than one. Therefore, the value of *K*_fs_ would decrease as the extraction temperature increases, which can result in decreased extraction efficiency as a similar situation reported by Chai and Pawliszyn [39]. Consequently, 60°C was the optimal choice as obtained in Figure 2(b), when considering the extraction temperature.

### 3.4. Effect of Extraction Time

As shown in Figure 2(c), we studied the SPME analyses run at selected time, the extraction efficiency of five analytes increased rapidly as extraction time from 10 min to 20 min, especially for GSM and *β*-cyclocitral, while a slow increase was observed for them between 20 and 40 min except GSM even declining, and the trend was tending towards stability after 40 min. However, the equilibrium time for this fiber maybe 30 min or more, but we desired shorter extraction time to maximize sample. Therefore, an extraction time 30 min was selected for experiments, and also this allowed the GC-MS analysis (25 min) to be performed nearly in the approximate time as HS-SPME procedure.

### 3.5. Effect of Desorption Time

As shown in Figure 2(d), desorption time (1, 2, 3, 5, and 7 min) profile is studied. Although their growth was inconsistent in the first five minutes, the peak area of five target compounds remained unchanged when desorption time is after 5 min. In other words, 5 min was enough for desorption. Thus, 5 min was selected as the optimal time.

### 3.6. Effect of Ionic Strength

The suitable salt addition could improve the transfer of analytes from the aqueous phase to the gaseous phase so this can result in a higher concentration of the odors in the headspace. Responses were calculated upon the condition of 5, 10, 15, 20, and 25% (w/v) ionic strength. As shown in Figure 2(e), the overall trend inclined to be horizontal in selected ionic strength, and, also, it was fairly clear that 25% (w/v) was most suitable for the extraction process, and this concentration of salt was selected for the future experiments.

## 4. Method Validation

The proposed method had been validated in terms of accuracy, linearity, LOD, %RSD, and recovery, and the relevant analytical parameters were shown in Table 2. To be more specific, linearity was studied by extracting the five odor standard solutions at five concentration levels, ranging from 5 to 100 ng L^−1^. Calibration curves showed adequate coefficients of correlation (*R*) higher than 0.999 with RSDs below 9.9% (*n* = 7); this showed satisfactory precision. The five odorants gave excellent responses to GC-MS detection. The LOD of these compounds were calculated on the basis of *S*/*N* = 3 in SIM mode at a low concentration and were below 1.3 ng L^−1^.

**Figure 3:**
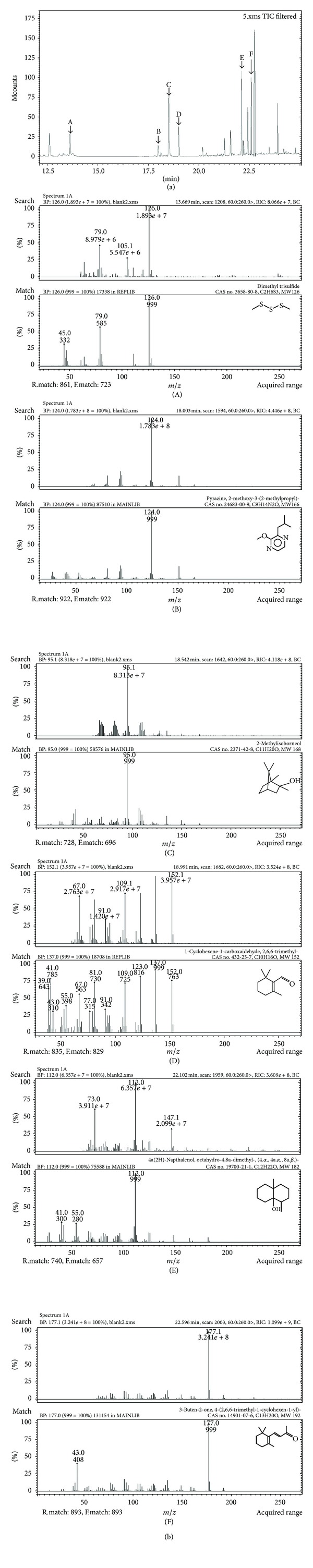
(a) MS-chromatogram of water sample (total ion current of the MS in the select ion mode) and (b) mass spectra of the six target compounds. Shown are (A) *DMTS*, (B) *IBMP*, (C) *2-MIB*, (D) **β**-cyclocitral, (E) *GSM*, and (F) **β**-ionone for both (a) and (b).

In addition, the method was applied to determine the target compounds in water samples from waterworks in Wuxi city. To confirm the validity of this method, we need to study the possible matrix effect in the water samples, and the result showed that there was no interfering peak from the sample matrix (Figure 3(a)). Moreover, according to the scan mode, the six target compounds in water samples can be identified and retrieved from MS spectrum library (Figure 3(b)). The recoveries of the five odors are between 83.2% and 112.3% in Table 3. Also, nine water samples from three waterworks were analyzed. The results are listed in Table 4, and, in conclusion, the proposed method has been proved to be rapid, sensitive, and reproducible enough to detect the trace compounds at nanogram-per-liter level.

## 5. Attenuation Experiment

The routine water samples often need a short-term for storage, because of the great quantity, the transportation delay that the samples are collected from sampling field to the lab, and time consuming on samples pretreatment. In addition, the musty odors GSM and 2-MIB are the required inspection items for drinking water in some countries, as in China and some other developing countries. Therefore, we conducted another experiment called attenuation or storage time experiment, to study the concentration decay subsequently.

To be more specific, two kinds of material vials had been applied to collect environmental samples, including glass vial for routine sampling and plastic vial (PET, polyethylene glycol terephthalate) which was convenient for specialists or citizens in case of some emergencies such as algae outbreak, ship leakage, flood, and earthquake, for the sake of collecting the typical samples. The water samples were obtained from Taihu Lake, Wuxi city. The mercuric chloride had been added to original water samples to inhabit microbial growth before the storage time experiment series. The result was shown in Table 5.

According to the result of analysis of variance calculated by SPSS 19.0, we did not find any statistically significant differences of the concentrations for both of GSM and 2-MIB during the storage time, and the *P* value was 0.92 and 0.98, respectively, for the plastic vial, whereas the glass vial was 0.69 and 0.80, respectively. Therefore, it is effective and reliable to detect GSM and 2-MIB in ten days if the water samples would be preserved in plastic or glass vial, and other required conditions, including sealed cap and 4°C in the dark.

## 6. Conclusion

A simple and sensitive method for simultaneous analysis of five odors in environmental water was developed by HS-SPME coupled to GC-MS, including GSM and 2-MIB, as well as DMTS, **β**-cyclocitral, and **β**-ionone; and it is more practical to detect trace odors in environmental water for future study, if modifying the original magnetic stirrer into a new one. Moreover, the storage time experiment indicated that the concentrations did not change significantly for GSM and 2-MIB if they were stored in canonical environment in ten days.

## Figures and Tables

**Table 1:** The CAS number, molecular weight, boiling point, and odor threshold of the six compounds.

Compounds	CAS number	Molecular formula	Molecular weight	Boiling point^a^ (°C)	OTC^c^ (ng L^−1^)
DMTS	3658-80-8	C_2_H_6_S_3_	126	177	10
IBMP	24683-00-9	C_9_H1_4_N_2_O	166	236	1
2-MIB	2371-42-8	C_11_H_20_O	168	210	9
*β*-Cyclocitral	432-25-7	C_10_H_16_O	152	215	1.9 × 10^4^
GSM	19700-21-1	C_12_H_22_O	182	270^b^/249	4
*β*-Ionone	14901-07-6	C_13_H_20_O	192	239^b^/263	7

^a^Calculated by EPISuit v.4.10 (2011) developed by the US EPA 2011, and boiling points by Stein and Brown method.

^b^This boiling point was obtained by EPISuit v.4.10.

^c^OTC: odor threshold concentration, detected by sensory and cited from Mallevialle [14] and Young et al. [6].

**Table 2:** The parameters of the MS scan function (SIM mode) for the determination of analytes.

Compounds	*t*_*R*_ (min)	Segment (min)	Selected ions	*R* ^b^	RSD% (*n* = 7)	LOD^e^ (ng L^−1^)
DMTS	13.669	12.1–14.0	126^a^, 79,111	0.9998	9.9^c^, 12.1^d^	1.3
IBMP	18.003	17.0–18.1	124^a^, 94,151	—	—	0.1
2-MIB	18.542	18.1–18.7	107^a^, 95,135	0.9995	4.9^c^, 5.9^d^	0.5
*β*-Cyclocitral	18.991	18.7–20.0	137^a^, 152,123	0.9990	4.4^c^, 6.7^d^	0.2
GSM	22.102	20.0–22.3	112^a^, 126,97	0.9990	8.2^c^, 8.9^d^	0.2
*β*-Ionone	22.596	22.3–25.0	177^a^, 91,135	0.9811	7.1^c^, 9.8^d^	0.4

^a^Quantitative ions (*m*/*z*).

^b^Calibration curves with compounds concentration: 5, 10, 20, 50, and 100 ng L^−1^.

^c^RSD: relative standard deviation, using IBMP as the internal standard. Compound concentration: 20 ng L^−1^.

^d^Without internal standard. Compound concentration: 20 ng L^−1^.

^e^LOD: limit of detection was calculated on the basis of *S*/*N* = 3, this value is a mathematical approximation.

**Table 3:** The concentration and recovery of earthy and musty odors in water samples (all samples were tested two times).

Compounds	Tap water			Deionized water		
	Concentration (ng L^−1^)	Recovery (%)		Concentration (ng L^−1^)	Recovery (%)	
		20 ng L^−1^	100 ng L^−1^		20 ng L^−1^	100 ng L^−1^
DMTS	7.8	92.8	95.4	2.4	91.7	90.2
2-MIB	6.4	104.3	92.0	2.5	110.1	93.5
*β*-Cyclocitral	1.2	109.8	94.3	n.d.	112.3	107.9
GSM	1.5	90.8	99.7	n.d.	107.0	104.1
*β*-Ionone	n.d.^a^	85.7	83.2	n.d.	86.2	88.3

^a^n.d. means below the lower-limit of the calibration range.

**Table 4:** The concentration of the five odors detected in waterworks from Wuxi city (all samples were test two times).

Compounds	Waterworks A^e^ (ng L^−1^)			Waterworks B (ng L^−1^)			Waterworks C (ng L^−1^)		
	A1^a^	A2^b^	A3^c^	B1^a^	B2^b^	B3^c^	C1^a^	C2^b^	C3^c^
DMTS	37.5	27.8	30.9	22.4	38.7	51.6	250.3	—	30.7
2-MIB	298.2^d^	9.8	4.0	104.6	3.9	5.9	1.6	4.2	1.1
*β*-Cyclocitral	338.8^d^	68.6	6.4	120.4	12.2	0.9	n.d.	n.d.	n.d.
GSM	n.d.	n.d.	n.d.	n.d.	n.d.	n.d.	n.d.	n.d.	n.d.
*β*-Ionone	112.9	n.d.	n.d.	98.2	n.d.	n.d.	n.d.	n.d.	n.d.

^a, b, c^Represent source water, product water, and tap water, respectively.

^d^The samples above the upper-limit of the calibration range were diluted twice before the further test.

^e^Waterworks A is located at Taihu Lake, Wuxi city.

**Table 5:** Result^a^ for water storage time (all samples were tested three times).

Storage time^b^ (*d*)	Linearity (*R*)		Plastic vial^c^ (ng L^−1^)		Glass vial^c^ (ng L^−1^)	
	GSM	2-MIB	GSM	2-MIB	GSM	2-MIB
0	0.9997	0.9999	108.26	109.36	98.62	101.17
1	0.9991	0.9993	95.32	107.33	92.59	107.88
2	0.9998	0.9998	98.32	94.02	94.62	89.61
3	0.9997	0.9934	105.62	107.04	98.63	95.17
4	0.9987	0.9995	104.85	96.13	96.86	95.86
5	0.9986	0.9995	108.26	106.07	97.31	94.69
6	0.9941	0.9998	98.98	109.36	95.26	94.51
7	0.9997	0.9987	105.24	97.01	96.54	96.18
10	0.9917	0.9974	96.13	105.95	98.05	91.74

^a^The concentration of target compounds in original water, GSM and 2-MIB, n.d., and 1.1 ng L^−1^, respectively.

^b^The 0 day means the day of sampling, 1 day means one day after 0 day, and so on.

^c^The 100 ng L^−1^ mixed standard of GSM and 2-MIB was added to both plastic and glass vial.

## Abbreviation

GSM: Geosmin
2-MIB: 2-Methylisoborneol
DMTS: Dimethyl trisulfide
IBMP: 2-Isobutyl-3-methoxypyrazine
CLSA: Closed-loop stripping analysis
LLME: Liquid-liquid microextraction
SBSE: Stir bar sorptive extraction
SPE: Solid-phase extraction
P&T: Purge and trap
HS-SPME: Headspace solid phase microextraction
GC-MS: Chromatography-mass spectrometry
LOD: Limit of detection
RSD: Relative Standard Deviation
OTC: Odor threshold concentration.
